# A MYBL2 complex for RRM2 transactivation and the synthetic effect of MYBL2 knockdown with WEE1 inhibition against colorectal cancer

**DOI:** 10.1038/s41419-021-03969-1

**Published:** 2021-07-07

**Authors:** Qian Liu, Lijuan Guo, Hongyan Qi, Meng Lou, Rui Wang, Boning Hai, Kailun Xu, Lijun Zhu, Yongfeng Ding, Chen Li, Lingdan Xie, Jing Shen, Xueping Xiang, Jimin Shao

**Affiliations:** 1 Department of Pathology & Pathophysiology, and Cancer Institute of the Second Affiliated Hospital, Zhejiang University School of Medicine, Hangzhou, China; 2 Zhejiang University Cancer Center, Key Laboratory of Disease Proteomics of Zhejiang Province, Key Laboratory of Cancer Prevention and Intervention of China National Ministry of Education, Zhejiang University School of Medicine, Hangzhou, China; 3 Key Laboratory of Pancreatic Disease of Zhejiang Province, First Affiliated Hospital, Zhejiang University School of Medicine, Hangzhou, China; 4 Department of Human Genetics, Zhejiang University School of Medicine, Hangzhou, China

**Keywords:** Cancer, Cancer

## Abstract

Ribonucleotide reductase (RR) is a unique enzyme for the reduction of NDPs to dNDPs, the building blocks for DNA synthesis and thus essential for cell proliferation. Pan-cancer profiling studies showed that RRM2, the small subunit M2 of RR, is abnormally overexpressed in multiple types of cancers; however, the underlying regulatory mechanisms in cancers are still unclear. In this study, through searching in cancer-omics databases and immunohistochemistry validation with clinical samples, we showed that the expression of MYBL2, a key oncogenic transcriptional factor, was significantly upregulated correlatively with RRM2 in colorectal cancer (CRC). Ectopic expression and knockdown experiments indicated that MYBL2 was essential for CRC cell proliferation, DNA synthesis, and cell cycle progression in an RRM2-dependent manner. Mechanistically, MYBL2 directly bound to the promoter of RRM2 gene and promoted its transcription during S-phase together with TAF15 and MuvB components. Notably, knockdown of MYBL2 sensitized CRC cells to treatment with MK-1775, a clinical trial drug for inhibition of WEE1, which is involved in a degradation pathway of RRM2. Finally, mouse xenograft experiments showed that the combined suppression of MYBL2 and WEE1 synergistically inhibited CRC growth with a low systemic toxicity in vivo. Therefore, we propose a new regulatory mechanism for RRM2 transcription for CRC proliferation, in which MYBL2 functions by constituting a dynamic S-phase transcription complex following the G1/early S-phase E2Fs complex. Doubly targeting the transcription and degradation machines of RRM2 could produce a synthetic inhibitory effect on RRM2 level with a novel potential for CRC treatment.

## Introduction

Colorectal cancer (CRC) is the third most common malignant tumor in both male and female, and the third leading cause of cancer death in the world [1]. By 2030, the global burden of CRC is expected to increase by 60% to more than 2.2 million new cases and 1.1 million deaths [2]. This indicates that further understanding of the mechanisms underlying CRC development is still urgently needed, which is the basis for development of novel therapeutic strategies for CRC treatment.

Ribonucleotide reductase (RR) is a unique enzyme for catalyzing the conversion of ribonucleotides (NDPs) to deoxyribonucleotides (dNDPs), which are the building blocks for DNA synthesis and thus essential for cell proliferation [3]. The holoenzyme of RR is composed of the large subunit RRM1 and the small subunit RRM2 or RRM2B, forming two types of RR, i.e., RRM1-RRM2 and RRM1-RRM2B, responsible for DNA replication and repair, respectively [4]. Pan-cancer mRNA expression profiling studies showed that the expression of RR subunits, especially RRM2, are upregulated in multi-types of cancers [5]. RRM2 plays an active role in tumor development and progression, and high RRM2 expression is associated with poorer patients outcomes in cancers [6, 7]. Inhibition of RR enzyme activity, as an important anticancer strategy, has been successfully used in clinical control of multiple solid and hematological malignancies [4].

The expression of RRM2 is rigorously regulated in response to the cell cycle regulation and DNA-damaging signals in normal cells. In cancers, the increased level of RRM2 is abnormally regulated mainly through transcription and degradation pathways, since there are extremely low copy number variation (CNV) and mutation rates in RRM2 gene [5]. The expression and activity of RR is exquisitely regulated during cell cycle progression. While the expression level of RRM1 is constant in actively proliferating cells, RRM2 expression is induced in G1 phases, peaks in S-phase, and is degraded in G2/M phase [8–10]. Therefore, RRM2 level controls the cell-cycle-dependent activity of RR for DNA synthesis and cell proliferation [5]. Among varieties of transcription factors (TFs) that participate in the cell cycle regulation, E2Fs and MYBL2-related complexes play a most important role in transcriptional regulation mainly in the G1 and S phases, respectively [11]. MYBL2, a member of the MYB family, is widely expressed in proliferative cells and crucial for the regulation of proliferation and differentiation. It is frequently overexpressed in several cancers and associated with poor patient prognosis, such as breast cancer [12], hepatocarcinoma [13], and colorectal cancer [14]. Downregulation of MYBL2 results in the inhibition of cell cycle progression and the promotion of apoptosis through multiple pathways [15]. WEE1 is involved in a RRM2 degradation pathway by inhibiting the activities of CDK1/2, which phosphorylate RRM2 leading to its ubiquitylation and degradation. Inhibition of WEE1 promotes the degradation of RRM2 through untimely phosphorylation and activation of CDK [16]. However, the detailed abnormally regulatory mechanisms for RRM2 expression in cancers and their implications in cancer treatment are still elusive.

In this study, we showed that the expression of MYBL2 was significantly upregulated in parallel with RRM2 in the cancer tissues of clinical CRC patients. MYBL2 associated with the newly identified partner TAF15 and MuvB components enhanced the malignancy by directly transcriptionally regulating RRM2 expression during the S-phase in CRC cells. Knockdown of MYBL2 significantly sensitized CRC cells to WEE1 inhibition in vitro and in vivo. Thus, we propose a new regulatory mechanism for RRM2 transcription by the S-phase MYBL2 complex in CRC cells and a novel potential synergistically therapeutic strategy by simultaneously inhibiting transcription and promoting degradation of RRM2 for CRC treatment.

## Results

### MYBL2 expression was upregulated in parallel with RRM2 level in clinical CRC patient samples

**Fig. 1:**
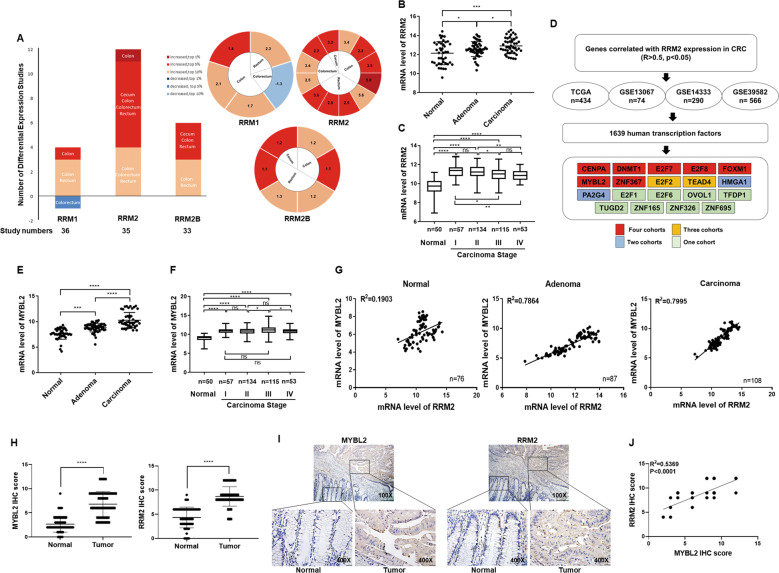
MYBL2 expression was upregulated in parallel with RRM2 level in clinical CRC patient samples. **A** The mRNA expression profiles (left) and the fold changes (right) of RRM1, RRM2, and RRM2B in CRC. All mRNA expression data of three RR subunit genes in CRC were downloaded from Oncomine database. DEGs were screened by “data type = mRNA, concept filter = cancers vs. normal, *P* < 0.05.” The *y*-axis of left panel presented the number of studies in which RRM1, RRM2, and RRM2B were differentially expressed according to the criteria. Fold changes shown in the color cells of the right panel were calculated by “cancer vs. normal” from the differentially expressed studies in the left panel. Dark red represented cancers in which the interested gene had a gene rank of the top 1% in the elevated-expression of the DEGs, and red and brick red marked top 5%, or 10%, respectively, while dark blue, blue, and light blue colors marked the top 1, 5, and 10% in the decreased-expression of the DEGs, respectively. Gene rank: genes are ranked by the *p*-value list. **B, C** The increased mRNA expression of RRM2 during CRC development. The mRNA expression data and clinicopathological information of CRC patients were downloaded from GSE20916 (**B**) and TCGA (**C**), respectively. **D** Screening of RRM2-correlated transcription factors (TFs). The expression correlation between RRM2 and all other genes in four CRC cohorts was measured by Pearson correlation coefficient. The human TFs that were positively correlated with RRM2 expression (R > 0.5, *p* < 0.05) were selected and listed in alphabetical order. **E, F** The increased mRNA expression of MYBL2 during CRC development. The mRNA expression data and clinicopathological information of CRC patients were downloaded from GSE20916 (**E**) and TCGA (**F**), respectively. **G** The expression correlation between MYBL2 and RRM2 in colorectal normal, adenoma, and carcinoma tissues, respectively. The mRNA expression data and clinicopathological information of CRC patients were downloaded from GEO (GSE20916, GSE8671, and GSE35896). **H** The protein expression level of MYBL2 and RRM2 determined by IHC in the paired cancer and adjacent normal tissues from 69 CRC patients. **I** Representative IHC images of MYBL2 and RRM2 in the CRC and adjacent noncancerous tissues (consecutive sections). Scale bars: 200 μm (100×), 25 μm (400×). **J** The correlation of IHC scores between MYBL2 and RRM2 in the 69 CRC tissues. ns, not signifucant; *p < 0.05; **p < 0.005, ***p < 0.001, ****p < 0.0001.

By analyzing all CRC data from Oncomine, we showed that the mRNA level of RRM2 was mostly increased among three RR subunits, ranking in the top 10% of the upregulated differentially expressed genes (DEGs) in 34.3% of the studies (12 of 35), in comparison with 11.1% (4 of 36) and 18.2% (6 of 33) for RRM1 and RRM2B, respectively. The RRM2 mRNA levels were significantly upregulated by 2.2–5.6-fold in cancerous vs. normal tissues, while the mRNA levels of RRM1 and RRM2B were changed less in these studies (Fig. 1A). Furthermore, the mRNA levels of RRM2 were increased both in colorectal adenomas and carcinomas (Fig. 1B) and maintained upregulations in all CRC TNM stages (Fig. 1C) compared to the adjacent normal tissues.

All genes which were significantly positively correlated with RRM2 in mRNA expression (r > 0.5, *p* < 0.05) were selected in the CRC patients from TCGA cohort and three GEO cohorts. By combined analyses with the TF database [17], 19 TFs were identified in the positively related genes, and among which, seven of them were commonly changed in the four cohorts of CRC patients, including MYBL2 (Fig. 1D). FOXM1 and E2F1, which have been shown to transcriptionally regulate RRM2 expression in prostate cancer [18], glioblastomas [19], and CRC [20], respectively, were also included. However, while FOXM1 was shared in all four cohorts, E2F1 was just positively correlated with RRM2 in one cohort of CRC. In comparison with adjacent noncancerous tissues, the mRNA expression levels of MYBL2 were increased both in colorectal adenomas and all stages of CRC in the TCGA and GEO cohorts (Figs. 1E, 1F). As expected, the expression correlation between RRM2 and MYBL2 was significantly strengthened in the colorectal adenoma and carcinoma tissues accordingly (Fig. 1G).

Finally, immunohistochemistry (IHC) staining showed that the protein expressions of both MYBL2 and RRM2 were significantly upregulated in the cancer tissues compared to their paired normal tissues in our cohort of 69 CRC patients (Figs. 1H, 1I) with a strong correlation coefficient between their expression levels (Fig. 1J).

### MYBL2 was essential for CRC cell proliferation, DNA synthesis, and cell cycle progression dependent on RRM2

**Fig. 2:**
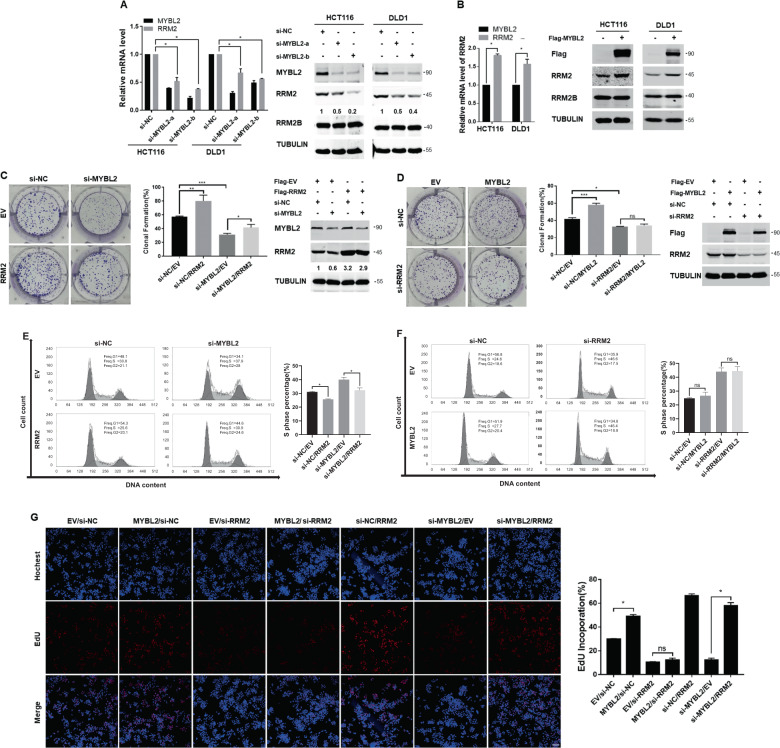
MYBL2 was essential for proliferation, cell cycle progression, and DNA synthesis of CRC cells in dependence on RRM2. **A, B** The effects of knockdown or overexpression of MYBL2 on the expression of RRM2 in HCT116 or DLD1 cells. The mRNA levels were analyzed by qRT-PCR (normalized by actin), and the protein levels were detected by western blotting analysis with antibodies against MYBL2, RRM2, RRM2B, and TUBULIN (as loading control), respectively. **C, D** The proliferation of DLD1 cells determined by clonal-formation assays after the indicated treatments (overexpression or knockdown). The efficiency of transfection or knockdown in DLD1 cells was determined by western blotting (right panels). **E, F** The cell cycle distributions of DLD1 cells was detected by FACS analysis after the indicated transfection or knockdown. **G** The DNA synthesis was measured by EdU incorporation assays in DLD1 cells after the indicated transfection or knockdown. Scale bars: 250 μm, unpaired Student’s *t*-test (two-tailed) was used to analyze the significance between different groups. ns, not significant; *p < 0.05; **p < 0.005, ***p < 0.001.

Gene expression manipulation analyses showed that while siRNA knockdown of MYBL2 significantly decreased the mRNA and protein expressions of RRM2 (Fig. 2A), ectopic expression of MYBL2 obviously increased the mRNA and protein expressions of RRM2 in CRC cells (Fig. 2B). The results indicated that MYBL2 upregulated RRM2 expression in CRC cells.

Clonal-formation assays showed that silencing of MYBL2 significantly inhibited the cell proliferation but RRM2 overexpression partially reversed the inhibition (Fig. 2C). On the other side, overexpression of MYBL2 promoted the cell proliferation whereas the combination with RRM2 knockdown abolished this effect (Fig. 2D, Fig. S3). Furthermore, FACS analyses showed that the exogenous expression of RRM2 partially remedied the S-phase arrest caused by MYBL2 knockdown (Fig. 2E), while MYBL2 overexpression did not save the S-phase arrest induced by silencing RRM2 (Fig. 2F). Finally, EdU incorporation assays showed that overexpression of MYBL2 improved DNA synthesis while knockdown of RRM2 abolished the effect, in comparison, RRM2 overexpression partially rescued the inhibition by silencing MYBL2 (Fig. 2G). These results indicated that MYBL2 promoted CRC cell proliferation in a RRM2-dependent way.

### MYBL2 activated the transcription of RRM2 gene by directly binding to its promoter in CRC cells

**Fig. 3:**
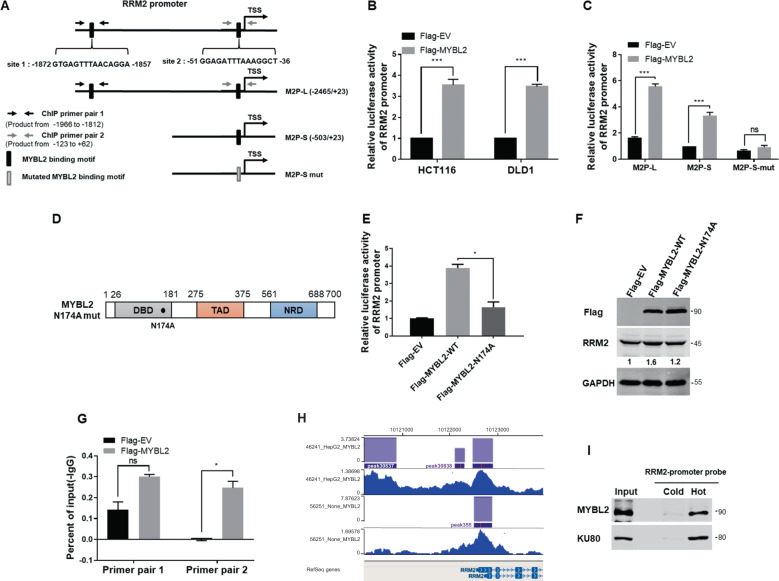
MYBL2 activated the transcription of RRM2 by directly binding to RRM2 promoter in CRC cells. **A** The schematic diagram for two MYBL2 biding motifs on RRM2 promoter and related truncated and site-mutated sequences. **B** Relative luciferase reporter activity of RRM2 promoter (−2465/+23) while co-transfected with MYBL2 expression plasmid for 48 h in HCT116 and DLD1 cells, respectively, pRL-SV40 as an internal control reporter. **C** Relative luciferase reporter activity of the truncated and mutated RRM2 promoters while co-transfected with MYBL2 expression plasmid for 48 h in DLD1 cells, pRL-SV40 as an internal control reporter. **D** The DNA-binding deficient mutation site in MYBL2 protein. **E** Relative luciferase reporter activity of RRM2 promoter (−2465/+23) while co-transfected with MYBL2 wild-type or N174A mutant. **F** The effects of overexpression of MYBL2 wild-type or N174A mutant on the expression of RRM2 in DLD1 cells. **G** Chromatin extracted from DLD1 cells was immunoprecipitated with the Flag or IgG antibodies, qRT-PCR were carried out on the immunoprecipitated DNAs using the specific primer pairs for the RRM2 promoter. **H** The ChIP-Seq data from Cistrome browser showed the binding of MYBL2 to RRM2 promoter in HepG2 cells. **I** Nuclear proteins obtained from DLD1 cells were pulled down by a non-biotin-labeled (cold) or biotin-labeled (hot) RRM2 promoter (−2465/+23) DNA probe, western blotting was used to detect the binding of MYBL2 or Ku80 (loading control). Unpaired Student’s *t*-test (2-tailed) was used to analyze the significance between different groups. ns, not significant; *p < 0.05; ***p < 0.001.

Two MYBL2 binding motifs exist in the predicted promoter region (from −2465 to +23) of RRM2 gene (Fig. 3A). Luciferase reporter assays showed that overexpression of MYBL2 significantly increased the RRM2 promoter activity, while the truncated promoter (delete site 1), especially the mutated promoter (delete both site 1 and 2) of RRM2 lost the reporter activity induced by the MYBL2 transfection in CRC cells (Figs. 3B, 3C). On the other hand, while transfection of the wild-type MYBL2 significantly induced RRM2 promoter reporter activity, the N174A mutant of MYBL2, which is important residue for maintaining its DNA-binding activity [21], lost the activity for RRM2 transactivation (Figs. 3D, 3E). Western blotting analysis also supported that the regulatory ability of the N174A mutant of MYBL2 for RRM2 expression was deceased compared with the wild-type (Fig. 3F).

Further, ChIP–PCR experiments demonstrated that MYBL2 was physically recruited to the RRM2 promoter on the site 2 in CRC cells (Fig. 3G), similar to the ChIP-Seq data of MYBL2 in the liver cancer HepG2 cells (Fig. 3H). Finally, DNA pull-down assays with the RR promoter probe validated that MYBL2 directly bound to the RRM2 promoter in the CRC cells (Fig. 3I). These data confirmed that MYBL2 was a transcriptional activator of RRM2 in CRC cells.

### The transcription of RRM2 was upregulated by a MYBL2 complex during cell cycle S-phase in CRC cells

**Fig. 4:**
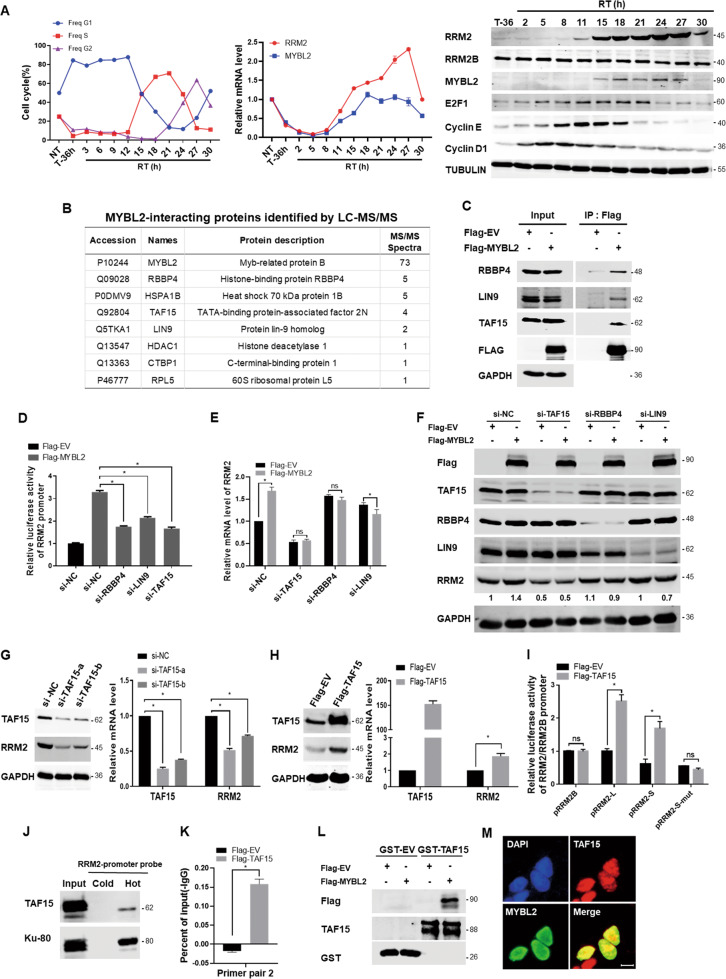
MYBL2-related complex contributed to RRM2 transcription during S-phase in CRC cells. **A** DLD1 cells were synchronized by 40uM lovastatin treatment for 36 h, and then released for analyses at the indicated time points. Left, FACS analyses of cell cycle phases (NT nontreatment, T treatment with lovastatin, RT remove treatment); Middle, qPCR for RRM2 and MYBL2; Right, western blotting for RRM2, RRM2B, MYBL2, E2F1, Cyclin E, Cyclin D1, and TUBULIN (as loading control). **B** DLD1 cells were transfected with empty vector (EV) or Flag-MYBL2 expression plasmid for 48 h, the cell lysates were co-immunoprecipitated with anti-Flag antibody, and MYBL2-interacting proteins were identified by LC-MS/MS. **C** The above MYBL2-interacting proteins were validated by western blotting with antibodies against RBBP4, LIN9, TAF15, FLAG, and GAPDH (as loading control). **D** The effects of knockdown of RBBP4, LIN9, or TAF15 on the activity of RRM2 promoter reporter (pRRM2-S) upregulated by MYBL2 transfection in DLD1 cells. **E, F** DLD1 cells were transfected with the siRNAs of negative control, TAF15, RBBP4, and LIN9, respectively, and then transfected with empty vector (EV) or MYBL2 expression plasmid for 48 h. qRT-PCR and western blotting were performed for analyses as indicated. **G, H** The effects of overexpression or knockdown of TAF15 on the mRNA and protein levels of RRM2 were analyzed by qRT-PCR (normalized by actin) and western blotting (GAPDH as loading control), respectively, in HCT116 cells. **I** Relative luciferase reporter activity of the truncated or mutated RRM2 promoter while co-transfected with TAF15 expression plasmid for 48 h in HCT116 cells, pRL-SV40 as an internal control reporter. **J** Nuclear proteins obtained from HCT116 cells were pulled down by a non-biotin-labeled (cold) or biotin-labeled (hot) DNA probe of RRM2 promoter (−2465/+23), western blotting was used to detect the biding of TAF15 and Ku80 (as binding control). **K** Chromatin extracted from HCT116 cells was immunoprecipitated with the Flag or IgG antibodies, qRT-PCR was carried out on immunoprecipitated DNAs using the primer pair2 as shown in Fig. 3A. **L** The whole lysates of HCT116 cells transfected with Flag-EV or Flag-MYBL2 were pulled down by GST-TAF15 or GST and then analyzed by immunoblotting. **M** HCT116 cells were plated into coverslip in six-well plate and then immunofluorescent staining was performed with antibody against TAF15 (red) or MYBL2 (green), scale bars: 10 μm.

DLD1 cells in different cell cycle phases were separated by FACS after synchronized by lovastatin for 36 h and then released, western blotting analyses showed that the expression of E2F1 increased in the G1 and early S phases and then started decreasing in the mid-S-phase, whereas the expression of MYBL2 and RRM2 initiated in the early S-phase, peaked at the mid-S-phase and then terminated in the eraly G2 phase (Fig. 4A). This dynamic expression profile suggested a sequential transcriptional regulation mode in which MYBL2 may regulate RRM2 transcription during the S-phase following the G1/early S-phase transcription factor E2F1. The expression of cyclin D1 and cyclin E in the G1/early S-phase correlative with E2F1 supported this mode. The expression of RRM2B, the other RR small subunit regulated by DNA repair pathways, did not change during the cell cycle progression, suggesting the specificity of MYBL2 for RRM2 transactivation.

To investigate the transcription regulatory mechanism, MYBL2-interacting proteins in DLD1 cells were co-immunoprecipitated and identified by LC-MS/MS (Fig. 4B), followed by western blotting validation (Fig. 4C). The results showed that MYBL2 interacted with TAF15 and the MuvB components LIN9 and RBBP4. For function analyses, luciferase reporter assays showed that silencing of TAF15, RBBP4, or LIN9 significantly reduced the activation of the RRM2 promoter reporter induced by MYBL2 transfection (Fig. 4D), suggesting that MYBL2 worked together with these proteins for RRM2 transcription. Furthermore, analyses at mRNA and protein expression levels showed that knockdown of TAF15 abolished the upregulation of RRM2 expression induced by MYBL2 more clearly than the other proteins (Figs. 4E, 4F).

The role of TAF15 in RRM2 transcription was further explored. Knockdown of TAF15 significantly decreased the mRNA and protein levels of RRM2 (Fig. 4G), while the overexpression of TAF15 increased the expression (Fig. 4H). Luciferase reporter assays showed that the overexpression of TAF15 significantly increased the transcriptional activity of the promoter of RRM2 but not RRM2B. Importantly, the mutation of the MYBL2 binding site 2 in RRM2 promoter completely disrupted the effect (Fig. 4I). Notably, both DNA pull-down (Fig. 4J) and ChIP–PCR (Fig. 4K) assays showed that TAF15 bound to the RRM2 promoter, although weaker than that of MYBL2 as shown in Fig. 3. Furthermore, GST pull-down analyses showed the interaction between TAF15 and MYBL2 again (Fig. 4L). Finally, immunofluorescence staining showed that MYBL2 co-localized with TAF15 in the cell nuclei (Fig. 4M). Thus, the above results suggested that TAF15 may participate in the MYBL2-driven RRM2 transcription by binding to the RRM2 promoter close to the MYBL2 binding site 2 and by interacted with MYBL2 which is known to form an S/G2 phase complex with the MuvB components.

### MYBL2 silencing enhanced the sensitivity of CRC cells to WEE1 inhibition in vitro and in vivo

WEE1 plays a key role in a RRM2 degradation pathway. MK-1775 is a WEE1 kinase inhibitor which currently in phase II clinical trials in combination with DNA-damaging agents [22]. We assumed that combined inhibiting transcription with promoting degradation of RRM2 might more efficiently downregulate the increased RRM2 level in cancer cells.

**Fig. 5:**
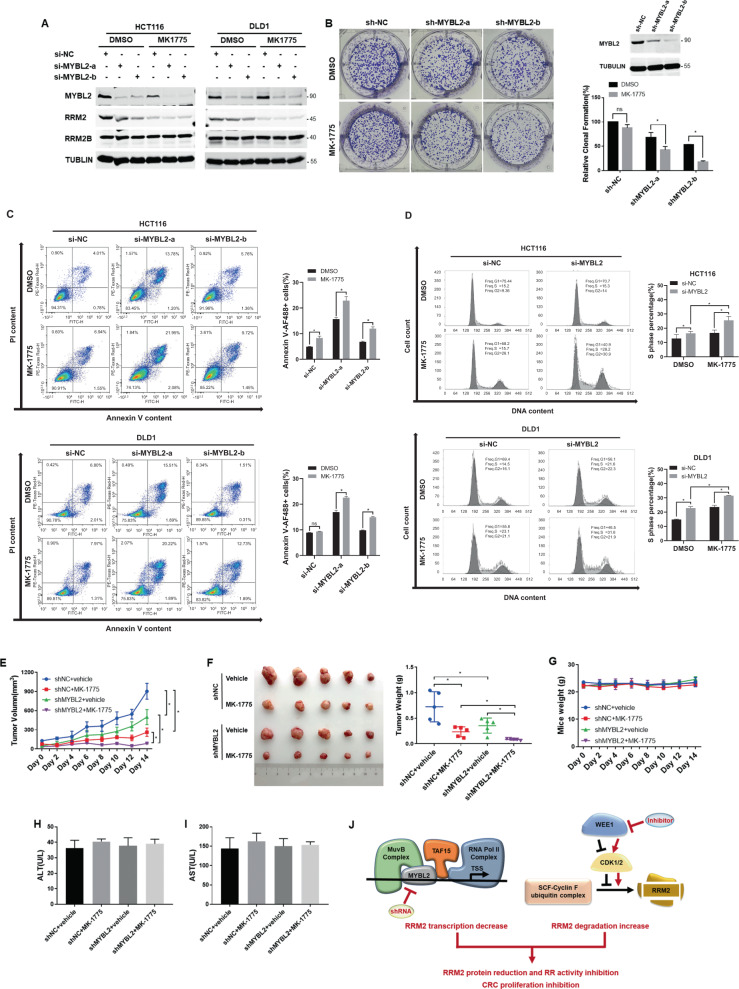
The effect of MYBL2 silencing combined with WEE1 inhibition on CRC cells and mice xenografts. **A** Western blotting analyses were performed to assess the levels of RRM2 in HCT116 and DLD1 cells treated with 500 nM MK-1775 for 24 h after knockdown MYBL2 for 48 h. **B** Clone-formation assays of HCT116 cells after shRNA-MYBL2 and treated with 100 nM MK-1775 for 1 week. The expression level of MYBL2 in HCT116 cells was shown by western blotting (upper right panel). **C, D** Flow cytometry assays were performed to assess cell apoptosis and cell cycle arrest in the DLD1 and HCT116 cells after treated with siRNA-MYBL2 for 48 h followed by exposure to 500 nM MK-1775 for 48 h. **E** The xenograft tumor volumes in each group of the mice were measured every 2-days following MK-1775 treatment. **F** The images (left) and weights (right) of the xenograft tumors of the mice were measured at day 15 after dissection. **G** Mouse body weights were measured every 2-days following the treatments. **H, I** The ALT and AST levels in sera of the mice. Data are presented as mean ± SEM, *n* = 5 mice. **J** Schematic representation of a MYBL2 complex for RRM2 transactivation and the synthetic effect of MYBL2 knockdown with WEE1 inhibition against colorectal cancer.

First, MK-1775 enhanced the downregulatory effect on the RRM2 protein levels by MYBL2 silencing in CRC cells (Fig. 5A). Second, the clonal-formation assays showed that silencing MYBL2 promoted the cell proliferation inhibition by MK-1775 treatment (Fig. 5B, S4), and the MTT assays showed that the ED50s of MK-1775 were reduced about 50% with the knockdown of WEE1 in CRC cells (Fig. S5). Third, MK1775-inducd cell apoptosis and cell cycle arrest were reinforced by the knockdown of MYBL2 in CRC cells (Figs. 5C, 5D). Finally, HCT116-shNC and HCT116-shMYBL2 cells were subcutaneously injected into nude mice, respectively, followed by oral gavage of MK1775 (Figs. 5E, 5F). Compared with the single-treatment groups, the combined treatment with shMYBL2 and MK1775 more significantly reduced the volumes and weights of the xenografts. While the tumor volume of the MK1775-treated group still showed a slow increase, the tumor volume of the doubly treated group hardly increased during the 14-day treatment (Fig. 5E). The doubly treatments achieved a significantly synergistic inhibitory effect on the tumor growth (Tumor Weight shNC + MK1775 group = 0.272 ± 0.098 g vs. Tumor Weight shMYBL2 + MK1775 group = 0.087 ± 0.015 g) (Fig. 5F).

Moreover, each group of the mice showed similar body weights during the treatments (Fig. 5G) and similar ALT and AST levels at the end of experiments (Fig. 5H, 5I), suggesting the treatments did not cause serious systemic damages in the mice. These results indicated that MYBL2 silencing combined with WEE1 inhibition produced a synergistic anticancer effect against the CRC cells and their mouse xenografts by doubly reducing RRM2 levels.

## Discussion

RR is a rate-limiting enzyme for DNA synthesis and thus a key determinant for cancer proliferation, one of the most important hallmarks of cancers [23]. The expression of RR subunit proteins, especially the RR activity controller RRM2, is significantly upregulated in multiple types of cancers. Inhibition of RR activity by small compound drugs such as gemcitabine and hydroxyurea has been demonstrated a useful treatment for cancers. However, the use of RR activity inhibitors can upregulate the expression of RRM1 or RRM2, leading to the drug resistance [24, 25]. Suppressing the expression of RRM2 by siRNA sensitizes cancer cells to both RR activity inhibitors and DNA-damaging drugs such as cisplatin. Therefore, further understanding of the abnormally regulatory mechanisms of RRM2 expression thereby RR activity in cancers is of important significance for cancer treatment.

In this study, by searching in cancer-omics databases, we showed that increased RRM2 expression involved a variety of TFs in CRC patients. Among them, the expression of MYBL2 was significantly upregulated in parallel with RRM2 in the cancer tissues of all studied CRC cohorts (Fig. 1). Ectopic expression and knockdown experiments indicated that MYBL2 was essential for CRC proliferation through increasing RRM2 expression in vitro and in vivo (Fig. 2, Fig. 5). Several TFs have been identified for regulating RRM2 transcription in different cancers, such as E2F1 in CRC [20], BRCA1 and E2F1 in glioblastoma [19], HPVE7 in cervical cancer [26], and FOXM1 in prostate cancer [18], and etc. [27, 28]. In this study, we mechanistically showed that during the cell cycle progression, following the G1/early S-phase-upregulated E2F1, MYBL2 expression was sequentially increased in S-phase and it might continually drive RRM2 transcription by directly binding to the RRM2 promoter in CRC cells (Figs. 3, 4).

Under physiological conditions, the DREAM complex, consisting of p130/107, DP1/2, E2F4/5, and MuvB core, represses cell cycle gene transcription during the G0 phase. When entering the cell cycle, the transcription of some G1/S genes is activated by E2F1-3-associated MuvB complex, and then MYBL2 and FOXM1 complexes sequentially activates the expression of G2/M genes to propel cell cycle progression [11]. The MuvB complex, also known as LIN complex, comprising LIN9, LIN37, LIN52, RBBP4, and LIN54, is critical for coordinating cell cycle gene expression by participating in the formation of DREAM, MMB (MYBL2-MuvB), or FoxM1-MuvB complex [11, 29]. MYBL2 interacts with MuvB complex through its C-terminus while the N-terminus is responsible for DNA binding [29]. In tumors, loss of DREAM and expression disorders of MYBL2, MuvB, and FOXM1 have been frequently found [11, 30].

In our study, using affinity purifications and mass spectrometry, we showed that MYBL2 interacted with the MuvB components LIN9 and RBBP4 and a new partner TAF15. Knockdown of these molecules, especially knockdown of TAF15, significantly inhibited RRM2 transactivation by MYBL2. TAF15, a noncanonical TAF [31], is known to associate with a distinct subpopulation of TFIID by directly binding to the C-terminus of RNA pol II and regulate gene expression [32–35]. However, the DNA-binding motif of TAF15 has not been identified so far, although a TAF15-binding motif in RNA (GGUAAGU) has been reported previously [36]. We showed that TAF15 associated with MYBL2 and bound to the RRM2 promoter, although weakly than MYBL2, to activate RRM2 transcription (Fig. 4C–M). Since no significant expression changes of TAF15 existed between the cancer and normal tissues of CRC (Fig. S2), probably MYBL2 dominated the S-phase RRM2 transcription in the complex in CRC cells.

There are two pathways for RRM2 protein degradation in cells: first, during mitosis/G1 phase, RRM2 is degraded by the Cdh1-APC complex that recognizes a KEN box motif at the RRM2 N-terminus [9]; second, during G2 phase, following CDK-mediated phosphorylation of T33, RRM2 is degraded via SCF (Cyclin F) ubiquitin ligase complexes [10]. The WEE1 kinase inhibits the activities of CDK1 and CDK2 through Y15 phosphorylation [37, 38], thus preventing the degradation of RRM2 by T33 phosphorylation, while WEE1 inhibition degrades RRM2 through untimely CDK activation [22]. Here, we showed that the combination of MYBL2 knockdown with MK-1775, a clinical phase II trial WEE1-inhibitory drug, synergistically inhibited CRC proliferation in vitro and xenograft growth in vivo with a low systemic toxicity (Fig. 5A-5I).

In summary, this study demonstrates that MYBL2 is a newly found important TFs for RRM2 transactivation in CRC proliferation, it functions by forming a dynamic S-phase complex with TAF15 and MuvB components to promote the malignant aggressiveness, and thus a potential novel target for CRC inhibition. Simultaneously targeting the transcription and degradation machines of RRM2 more significantly decreases the protein level, which could be a new therapeutic strategy for more effective treatment of CRC, in addition to conventional inhibition of RR enzymatic activity, or the double downregulation of RRM2 level could promote CRC more sensitive to the enzyme activity inhibition (Fig. 5J).

## Materials and methods

### Reagents and antibodies

Antibodies against RRM2(sc-398294), RRM2B(sc-10840), LIN9(sc-398234), and Cyclin E(sc-247) were from Santa Cruz Biotech (Santa Cruz, CA). The MYBL2(ab12296), TAF15(ab134916), RBBP4(ab79391), E2F1(ab179445), and Ku80(ab79391) antibodies were from Abcam (Abcam, UK). The Tubulin (ER130905), GAPDH(EM1101), and Cyclin D1(ET1601-31) antibodies were from HuaBio (HuaBio, China). The Flag antibody (F1804-50UG) was from Sigma (Sigma–Aldrich, USA). Lovastatin (T1207) was from Topscience (Topscience, China). MK1775(S1525) was from Selleck (Selleck, China).

### Clinical samples and immunohistochemistry (IHC)

The fresh paired cancer and para-cancer normal tissues, and formalin-fixed, paraffin-embedded (FFPE) cancer tissues of clinical CRC patients were obtained from the Second Affiliated Hospital of Zhejiang University School of Medicine. The collection and using of the samples were performed according to the ethical standards formulated in the Declaration of Helsinki. Written informed consent was obtained from each patient, and the study was approved by the ethics committee of the Zhejiang University School of Medicine, China. The clinicopathological characteristics of the clinical specimens are shown in Table S1 and S2.

The IHC staining was performed with Envision detection system (Dako, Denmark) in FFPE CRC tissues. The primary antibodies included anti-RRM2 (ab57653, abcam) and anti-MYBL2-pT487(ab76009, abcam) [39]. The IHC score for slides were determined as previously described [40, 41].

### Cell lines, transfection, and treatments

The human CRC cell lines DLD1, HCT116, and SW480 were obtained from the American Type Culture Collection (ATCC, Manassas, VA) and maintained in RPMI 1640 medium. 293FT cells was cultured in DMEM medium. All cells were cultured at 37 °C with 5% CO_2_ and culture media contained extra 10% fetal bovine serum, 100 μg/mL streptomyc, and 100 units/mL penicillin. CRC cell lines were identified by STR profiling, and mycoplasmas were tested every six months.

Cells were transfected with plasmids using X-tremeGENE HP DNA transfection reagent (Roche, USA) and transfected with siRNAs using Lipofectamine RNAiMAX Reagent (Invitrogen, USA) according to the manufacturers’ instructions. To synchronize cell cycle, DLD1 cells were treated with 60 uM lovastatin for 36 h. For the knockdown of RBBP4 and LIN9, we obtained the sequences of siRNAs according to literatures [42, 43], while others were listed in Table S3.

For lentiviral transduction, the short hairpin RNAs (shRNA) targeting MYBL2 and nontarget control (shNC) were purchased from Hanbio (Shanghai, China) and used to establish stable-transfected HCT116 cell lines. The stable cell lines were treated with 1 μg/mL puromycin selection after virus infection.

### Immunofluorescence

HCT116 cell layers on glass coverslips were fixed for 15 min by 4% paraformaldehyde at 4 °C. Then, we used PBS containing 0.2% Triton X-100 permeabilizing cells for 20 min and blocking with PBS containing 1% BSA and 0.5% goat serum for 2 h at 37 °C. The cells were incubated with mouse anti-MYBL2(1:50) and rabbit anti-TAF15(1:100) antibodies, and following probed with corresponding secondary antibodies for 1 h at 37 °C. Finally, we stained the nuclei with DAPI for 15 min. The slides were visualized by a microscope.

### Quantitative real-time PCR

Total RNA was isolated from cells using RNAsio Plus (TaKaRa, Japan). Reverse transcription was performed with the PrimeScript RT reagent kit (TaKaRa, Japan). Quantitative real-time PCR (qRT-PCR) was achieved using SYBR® Premix Ex TaqTM (TaKaRa, Japan) on a LightCycler^®^480 System (Roche Diagnostics). Gene expressions were normalized to actin levels as an internal control. The sequences of the qRT-PCR primers are listed in Table S4. All experiments were carried out in triplicate.

### Western blotting

Cells were collected by centrifugation and washed twice with phosphate-buffered saline (PBS) and then lysed in in RIPA lysis buffer (Millipore, Germany), which was contained complete protease inhibitor (Roche) and phosphatase inhibitor (Roche). Whole-cell extracts lysed from treated cells were obtained by centrifugation, and the protein concentration was measured by the Bradford method (Bio-Rad, CA). The extracts were separated on SDS-PAGE and transferred to nitrocellulose membranes (Whatman, Maidstone, UK), which were incubated with dilutions of primary antibodies followed by incubation with IRDye 800CW or IRDye 680-conjugated secondary antibodies, and then visualized on the Odyssey Infrared Imaging System (LI-COR Biosciences, Lincoln, NE). All experiments were repeated three times and representative results were shown.

### Flow cytometry

3 × 10^5^ DLD1 or HCT116 cells were seeded onto 6 well plates, incubated for 24 h at 37 °C, and treated as indicated. For cell cycle analysis, cells were trypsinized, resuspended and fixed with 70% ethanol at −20 °C for at least 1 h. Before analysis, the cells were resuspended in PBS containing 10 mg/mL RNaseA (Multi sciences, China) and 50 mg/mL propidium iodide (PI; Multi sciences, China) for at least 30 min. For apoptosis analysis, the supernatant was transferred to eppendorf tubes and cells were trypsinized and then collected to supernatant and washed twice with cold PBS, followed by staining with Annexin V-FITC/PI (Multi sciences, China) for 15 min. PI- and Annexin V-stained cells were analyzed immediately on a ACEA NovoCyteTM (ACEA Biosciences, USA) or Cytomic FC 500MCL (BECKMAN COULTER, USA).

### Luciferase reporter assays

1 × 10^5^ DLD1 or HCT116 cells were seeded onto 24-well plates per well. The next day, the cells were co-transfected with 0.25 μg firefly luciferase reporter constructs, 0.25 ug TFs constructs and 10 ng pRL-SV40 Renilla luciferase reporter plasmids for 48 h. The pRL-SV40 plasmid was used to normalize the transfection efficiency. The luciferase activities were detected by luminometer (LB9507, Berthold Technologies, Bad Wildbad Germany) according to the Dual-Luciferase^®^ Reporter Assay System (E1910, Promega) technical manual. All results are representative of at least three independent experiments.

### DNA pull-down assays

The DNA pull-down assay method was modified from a published protocol [44]. Briefly, DLD1 cells were harvested by trypsin and the nuclear proteins were extracted by Nucleoprotein Extraction Kit (Sangon Biotech, China). The DNA probes, covering from −2465 to +23 of the human RRM2 promoter, were amplified by PCR with the primers (the reverse primer was labeled with biotin) and purified using a cycle purified kit (Omega, China). The biotinylated DNA probes were incubated with Pierce™ Streptavidin Agarose (Invitrogen) in binding buffer (5 mM Tris-HCl pH 7.5, 500 nM EDTA, 50 mM NaCl) overnight and washed three times. The beads were then added to the nuclear proteins and incubated for 4 h at 4 °C and washed with wash buffer (25 mM HEPES pH 7.5, 20% glycerol, 0.4% Triton X-100, 0.5 mM EDTA, and 150 mM NaCl) for five times. Finally, protein loading buffer was added to the precipitates, boiled for 5 min. Proteins pulled down by the DNA probes were separated on 10% SDS-PAGE and analyzed using immunoblotting.

### Chromatin immunoprecipitation (ChIP)

DLD1 cells were transfected with Flag-MYBL2 or Flag-TAF15 for 48 h, then cross-linked with 1% formaldehyde and sonicated DNA to ~200 bp. The supernatants obtained by centrifugation was immunoprecipitated overnight by the antibody against FLAG or normal rabbit IgG at 4 °C. Chromatin–protein-antibody complexes were isolated using protein A/G plus Magnetic agarose beads (Millipore). The crosslinking was reversed by heating and genomic DNA fragments were purified and analyzed by qRT-PCR using the two primer pairs for the RRM2 promoter: the site 1, 5′-TACGCATCTTTCGGCGTCTT-3′ (forward), 5′-AAAACCCTCGTTTCGGTTGC-3′ (reverse); and the site 2, 5′- GAGGCATGGCACAGCAA-3′ (forward), 5′-AGCAAGCTTGAGTGACCCAT-3′ (reverse). The results are representative of at least three independent experiments.

### Co-immunoprecipitation (Co-IP), LC-MS/MS, and GST pull down assays

DLD1 cells were transfected with Flag-MYBL2 for 48 h and lysed by IP lysis Buffer (Beyotime, China). The cell lysates were then co-immunoprecipitated by ANTI-FLAG^®^ M2 Magnetic Beads (Sigma–Aldrich, USA), followed by LC-MS/MS or western blotting analyses. Five percent of the cell lysate for each co-immunoprecipitation was used for the input control.

The expression plasmid for GST-TAF15 recombinant protein was constructed in pGEX-4T3 vector. The proteins were expressed in Escherichia coli Rosetta strain (Transgene). The GST pull-down assays were performed according to the previous publication [40].

### Plate clone-formation assays

1 × 10^3^ DLD1 or HCT116 cells were plated in six-well plates. After treatment and culture for 10 days, cells were washed twice with PBS and cross-linked with 4% formaldehyde for 30 min, and then were stained with the crystal violet staining solution for 15 min. The colony formation efficiency = (number of colonies/number of cells inoculated) × 100%. ImageJ software (National Institutes of Health, USA) was used to count the number of clones.

### EdU incorporation assays

DNA synthesis was analyzed by the Cell-Light™ EdU Apollo^®^567 In Vitro Imaging Kit (RiboBio Co., China) according to the instructions. Images of the cells were captured with a fluorescence microscope (Nikon, Tokyo, Japan). The number of EdU positive cells were counted by ImageJ software.

### Cancer cell growth inhibition assays

HCT116 and SW480 Cells with or without WEE1 knockdown by siRNA were seeded into 96-well tissue culture plates at 1,500 cells per well for 24 h, then two-fold dilutions of MK-1775 by from 0.01172 to 1.5 uM were added to the plates. After culturing for 7 or 10 days, cell growth was determined by MTT assays, and the results were measured at 490 nm.

### Nude mouse tumor xenograft experiments

The BALB/C nude mice (male, 4 weeks old) were subcutaneously injected into the right armpit with HCT116-shNC or HCT116-shMYBL2 cells (3 × 10^6^ cells per mouse, 10 mice per cell type). When the tumor was growing up for 1 week, mice were randomly assigned to two groups for each cell type. One group received 0.5% w/v methylcellulose (0.1 ml/10 g body weight) by oral gavage once a day for 14 days, while another group received 60 mg/kg MK-1775 in 0.5% w/v methylcellulose (0.1 ml/10 g body weight) by oral gavage once a day for 14 days. The long diameter (*a*) and short diameter (*b*) of the tumors were measured by caliper, and then the volume (*V*) was calculated as 1/2*a* × *b* [2]. Mice were sacrificed on day 15, 24 h after the last dose. The tumors were harvested, weighted and photographed. The ALT and AST levels in the mouse sera were measured by Zhejiang Chinese Medical University Laboratory Animals Research Center. All animal procedures were approved by Laboratory Animals Welfare Ethics Review Committee of Zhejiang University (ZJU20170522).

### Statistical analysis

All results were presented as the means ± SD of three independent experiments. Student’s *t*-tests and one-way ANOVA were used to analyze differences in expression among the groups. Pearson’s χ2 test was used to evaluate the correlations between the expression of RRM2 and TFs in CRC datasets. *P-*values < 0.05 were considered significant, and statistical analyses were performed using GraphPad prims 7.

## Supplementary information

Supplemental tables

Supplemental Figure Legends

Supplemental Figure 1

Supplemental Figure 2

Supplemental Figure 3

Supplemental Figure 4

Supplemental Figure 5

## Data Availability

The datasets analyzed during the current study are available in the Oncomine (https://www.oncomine.org/), The Cancer Genome Atlas (https://www.cancer.gov/) or Gene Expression Omnibus (https://www.ncbi.nlm.nih.gov/geo/) under the accession number GSE20916, GSE8671, and GSE35896.
