# Supplementary material for: A MYBL2 complex for RRM2 transactivation and the synthetic effect of MYBL2 knockdown with WEE1 inhibition against colorectal cancer
Source: Cell Death Dis. 2021 Jul 7;12(7):683. doi: 10.1038/s41419-021-03969-1 (PMC8263627; doi:10.1038/s41419-021-03969-1)
Supplement: Supplementary file 1 — Supplemental tables [file 41419_2021_3969_MOESM1_ESM.docx]

Table S1 The clinical information of colorectal cancer tissue for Western blot.

| ID | Sex | Age | Tissue | Primary Tumor | Chemoradiotherapy | TNM  stage |
| --- | --- | --- | --- | --- | --- | --- |
| 10553795 | F | 77 | Rectum | No | Yes | III |
| 10589848 | M | 50 | Rectum | No | Yes | III |
| 10605282 | F | 51 | Colon | No | Yes | III |
| 10611161 | F | 54 | Rectum | No | Yes | III |
| 10617914 | M | 70 | Colon | No | Yes | IV |
| 10619274 | M | 33 | Rectum | No | Yes | III |
| 10628533 | M | 63 | Colon | No | Yes | III |
| 10631462 | F | 89 | Rectum | No | Yes | IV |
| 00450101 | F | 78 | Colon | No | Yes | III |
| 00729692 | F | 26 | Colon | No | Yes | III |
| 08176237 | F | 36 | Rectum | No | Yes | III |
| 09046536 | M | 63 | Colon | No | Yes | IV |

Table S2 Clinico-pathologic parameters of colorectal cancer patients for IHC

| Characteristics | Number(%) |
| --- | --- |
| Gender |  |
| Male | 38(55) |
| Female | 31(45) |
| Age |  |
| Mean(Range) |  |
| <65 | 35(51) |
| >65 | 34(49) |
| Tumor size |  |
| <5cm | 31(45) |
| ≥5cm | 38(55) |
| Depth of invasion |  |
| T1 | 2(3) |
| T2 | 7(10) |
| T3 | 40(58) |
| T4 | 17(25) |
| Lymph node metastasis |  |
| N0 | 44(64) |
| N1 | 18(26) |
| N2 | 7(10) |
| Distant metastasis |  |
| M0 | 61(88) |
| M1 | 8(12) |
| TNM stage |  |
| I | 6(9) |
| II | 35(51) |
| III | 22(32) |
| IV | 6(9) |

Table S3 The sequences of siRNAs

| Gene | Sense | Antisense |
| --- | --- | --- |
| si-MYBL2-a | GCCAUGGACCAAAGAGGAATT | UUCCUCUUUGGUCCAUGGCTT |
| si-MYBL2-b | CCUUCCUGGAUUCCUGUAATT | UUACAGGAAUCCAGGAAGGTT |
| si-TAF15-a | GGACAGAACUACAGCGGUUTT | AACCGCUGUAGUUCUGUCCTT |
| si-TAF15-b | GCCAGCAACCAUAUAAUAATT | UUAUUAUAUGGUUGCUGGCTT |
| si-RRM2-a | CCCAUCGAGUACCAUGAUATT | UAUCAUGGUACUCGAUGGGTT |
| si-RRM2-b | CGUCGAUAUUCUGGCUCAATT | UUGAGCCAGAAUAUCGACGTT |
| si-WEE1-a | GCUGAUGCUAUAAGUGAAATT | UUUCACUUAUAGCAUCAGCTT |
| si-WEE1-b | GUCCCGGUAUACAACAGAATT | UUCUGUUGUAUACCGGGACTT |
| si-NC | CUUACGCUGAGUACUUCGATT | UCGAAGUACUCAGCGUAAGTT |

Table S4 The sequences of the qRT-PCR primers

| Gene | Forward primer:5’-3’ | Revers primer:5’-3’ |
| --- | --- | --- |
| RRM2 | GCAGCAAGCGATGGCATAGT | GGGCTTCTGTAATCTGAACTTC |
| RRM2B | TAAACAGGCACAGGCTTCCT | CGCTCCACCAAATTTTCATT |
| MYBL2 | CTTGAGCGAGTCCAAAGACTG | AGTTGGTCAGAAGACTTCCCT |
| TAF15 | ACAGCGGTTACTCCAGTTATGG | CCATGTTTTGCTGCTGTCCC |
| ACTIN | TGGAGAAAATCTGGCACCACACC | GATGGGCACAGTGTGGGTGACCC |
