# Supplementary material for: A MYBL2 complex for RRM2 transactivation and the synthetic effect of MYBL2 knockdown with WEE1 inhibition against colorectal cancer
Source: Cell Death Dis. 2021 Jul 7;12(7):683. doi: 10.1038/s41419-021-03969-1 (PMC8263627; doi:10.1038/s41419-021-03969-1)
Supplement: Supplementary file 2 — Supplemental Figure Legends [file 41419_2021_3969_MOESM2_ESM.docx]

**Supplemental Figure Legends**

**Supplemental Figure 1 Representative graph of the protein expression of RRM2 in colorectal cancer patients.** The protein level of RRM2 was detected by western blot in 12-paired cancer and para-cancer normal tissues of clinic CRC samples.

**Supplemental Figure 2 The mRNA expression level of TAF15 in colorectal cancer.** The mRNA expression level of TAF15 in colorectal cancer by Gene Expression Profiling Interactive Analysis (GEPIA, <http://gepia.cancer-pku.cn/>).

**Supplemental Figure 3 The knockdown efficiency of RRM2 siRNA in DLD1 cells.** The protein and mRNA expression level of RRM2 were detected in DLD1 cells that were transfected with different siRNA for 48 hours.

**Supplemental Figure 4 Silencing MYBL2 promoted the cell proliferation inhibition by MK1775 treatment in SW480 cells.** (A) Clone-formation assays of SW480 cells after shRNA-MYBL2 and treated with 200nM MK-1775 for 10 days. (B) The expression level of MYBL2 in SW480 cells was shown by western blot.

**Supplemental Figure 5 The knockdown of WEE1 increased the sensitivity to MK-1775 in colorectal cancer cells.** (A) qPCR was performed to assess the efficiency of WEE1 knockdown by siRNA in HCT116 or SW480 cells. (B) MTT assay was performed to assess the ED50 of MK-1775 in HCT116 or SW480 cells with or without the knockdown of WEE1 by siRNA.
