# Supplementary figures and images for: A MYBL2 complex for RRM2 transactivation and the synthetic effect of MYBL2 knockdown with WEE1 inhibition against colorectal cancer
Source: Cell Death Dis. 2021 Jul 7;12(7):683. doi: 10.1038/s41419-021-03969-1 (PMC8263627; doi:10.1038/s41419-021-03969-1)

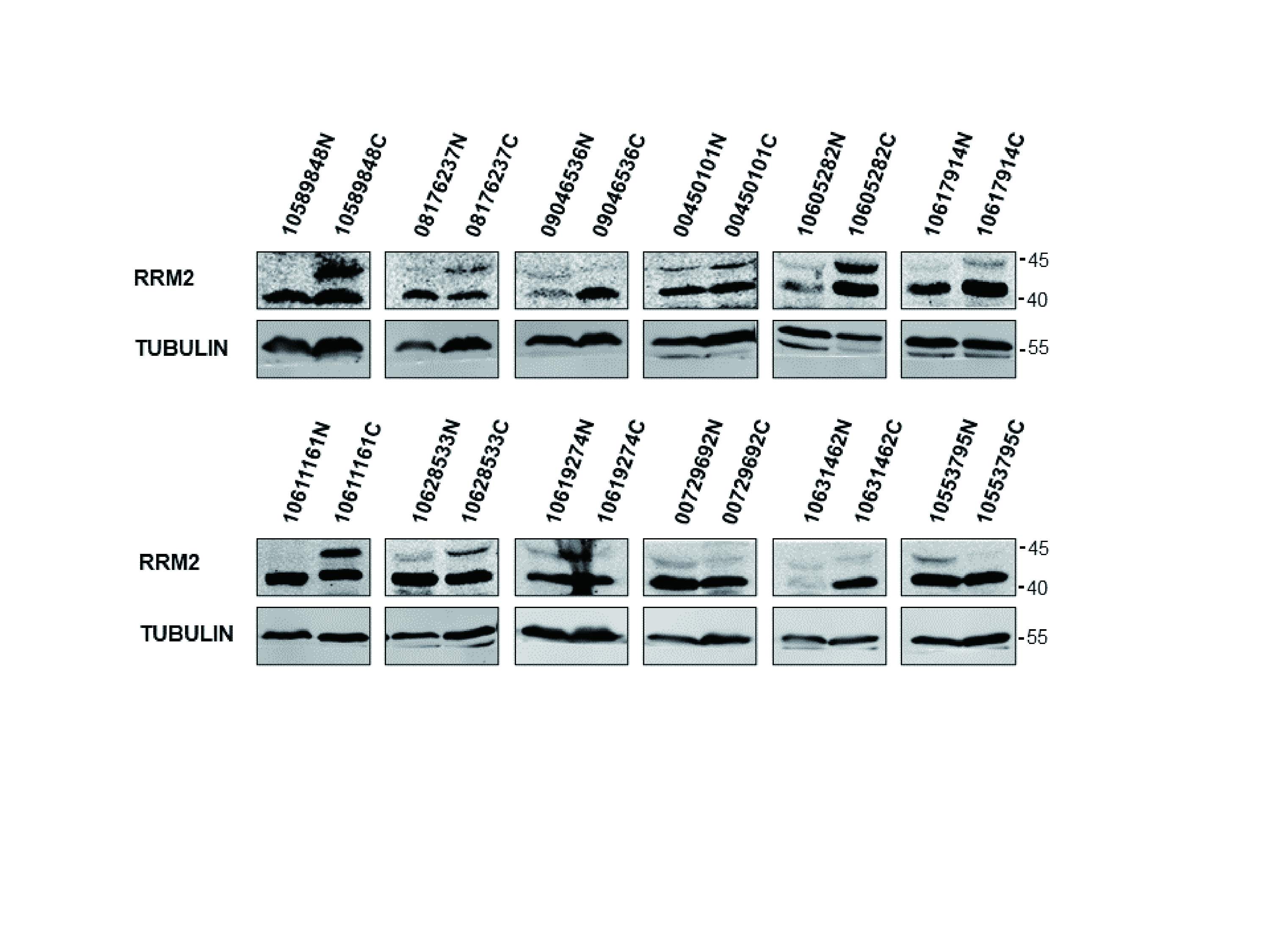

Supplement: Supplementary file 3 — Supplemental Figure 1 [file 41419_2021_3969_MOESM3_ESM.tif]

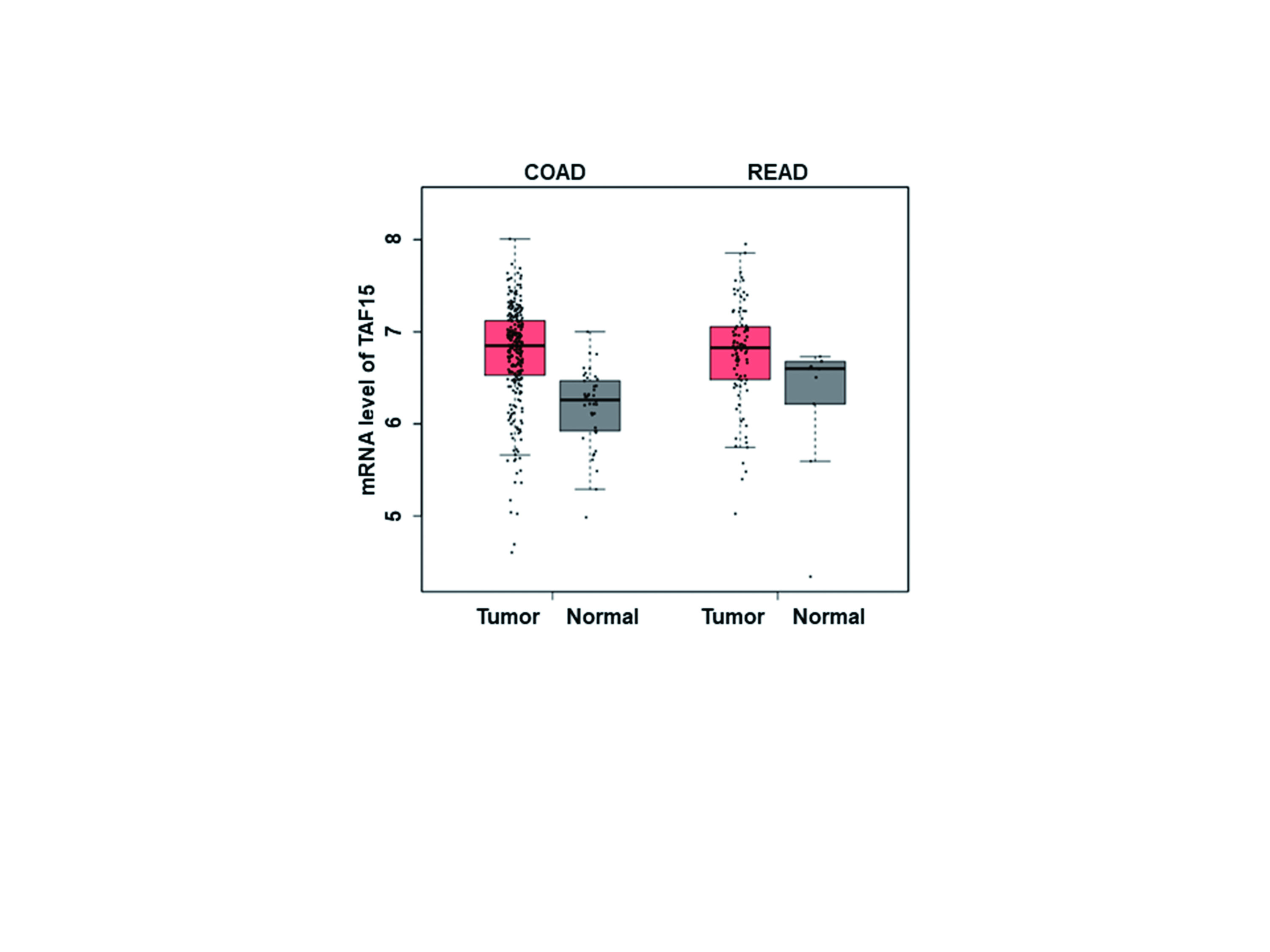

Supplement: Supplementary file 4 — Supplemental Figure 2 [file 41419_2021_3969_MOESM4_ESM.tif]

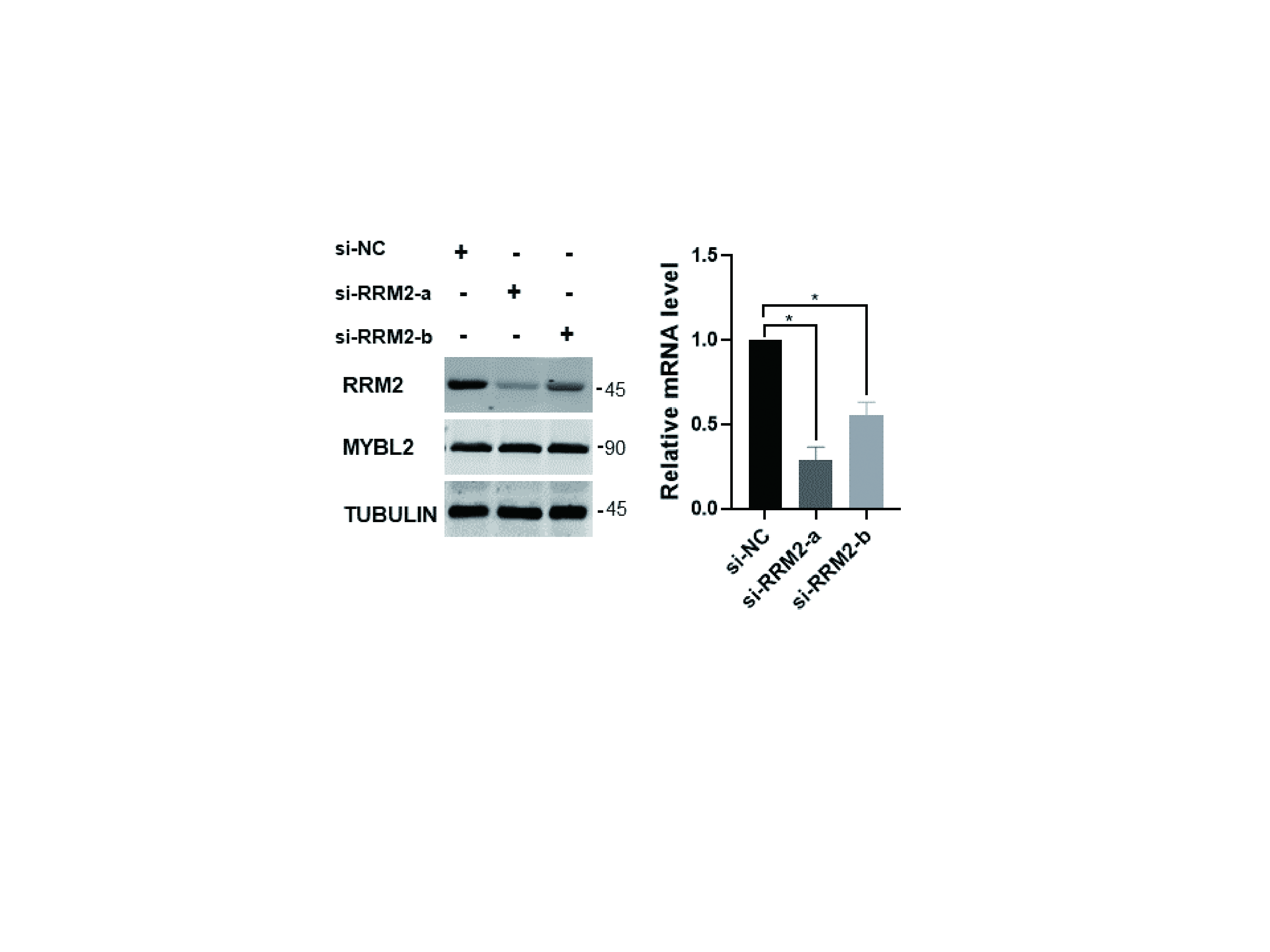

Supplement: Supplementary file 5 — Supplemental Figure 3 [file 41419_2021_3969_MOESM5_ESM.tif]

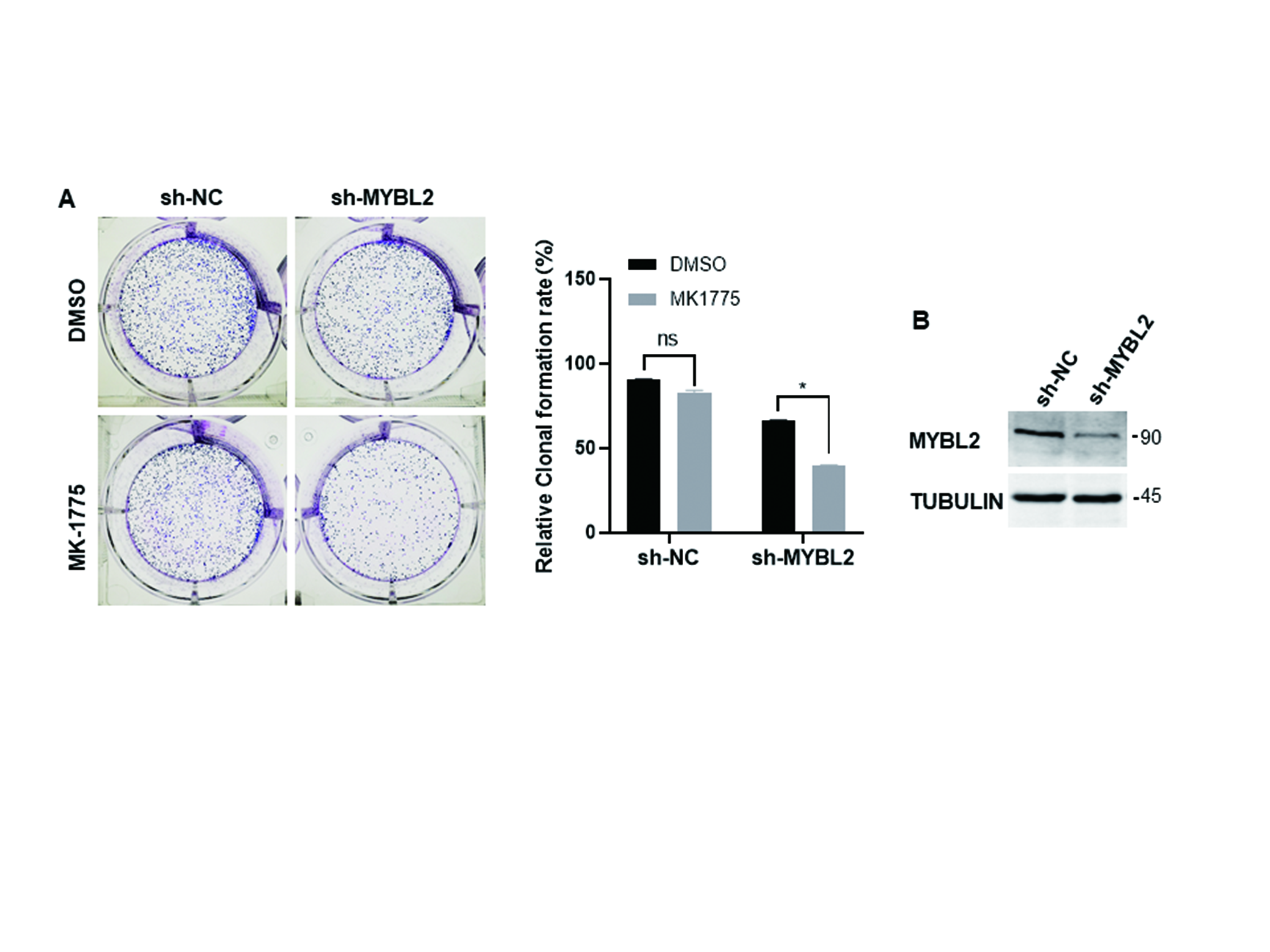

Supplement: Supplementary file 6 — Supplemental Figure 4 [file 41419_2021_3969_MOESM6_ESM.tif]

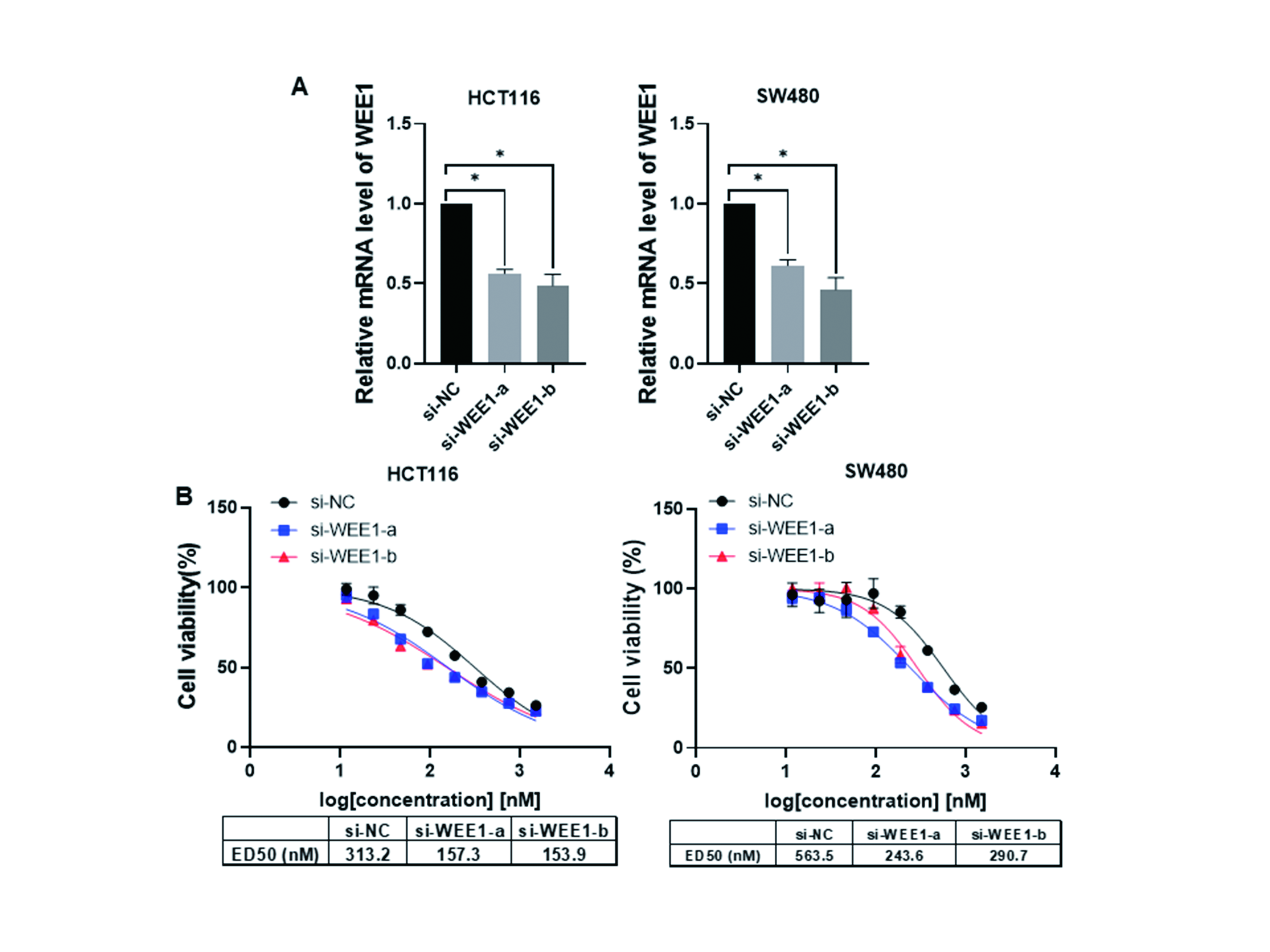

Supplement: Supplementary file 7 — Supplemental Figure 5 [file 41419_2021_3969_MOESM7_ESM.tif]
